# Inhibitory Effect of Vascular Endothelial Growth Factor on the Slowly Activating Delayed Rectifier Potassium Current in Guinea Pig Ventricular Myocytes

**DOI:** 10.1161/JAHA.117.007730

**Published:** 2018-01-26

**Authors:** Zhenhao Lin, Wenlu Xing, Chuanyu Gao, Xianpei Wang, Datun Qi, Guoyou Dai, Wen Zhao, Ganxin Yan

**Affiliations:** ^1^ Department of Cardiology Zhengzhou University People's Hospital Zhengzhou Henan China; ^2^ School of Pharmaceutical Sciences Zhengzhou University Zhengzhou Henan China; ^3^ Main Line Health Heart Center Lankenau Institute for Medical Research Wynnewood PA

**Keywords:** action potential, arrhythmia, phosphatidylinositol 3‐kinase, potassium channels, vascular endothelial growth factor, Growth Factors/Cytokines, Electrophysiology, Arrhythmias, Cell Signalling/Signal Transduction, Ion Channels/Membrane Transport

## Abstract

**Background:**

Vascular endothelial growth factor (VEGF) exerts a number of beneficial effects on ischemic myocardium via its angiogenic properties. However, little is known about whether VEGF has a direct effect on the electrical properties of cardiomyocytes. In the present study, we investigated the effects of different concentrations of VEGF on delayed rectifier potassium currents (I_K_) in guinea pig ventricular myocytes and their effects on action potential (AP) parameters.

**Methods and Results:**

I_K_ and AP were recorded by the whole‐cell patch clamp method in ventricular myocytes. Cells were superfused with control solution or solution containing VEGF at different concentrations for 10 minutes before recording. Some ventricular myocytes were pretreated with a phosphatidylinositol 3‐kinase inhibitor for 1 hour before the addition of VEGF. We found that VEGF inhibited the slowly activating delayed rectifier potassium current (I_K__s_) in a concentration‐dependent manner (18.13±1.04 versus 12.73±0.34, n=5, *P*=0.001; 12.73±0.34 versus 9.05±1.20, n=5, *P*=0.036) and prolonged AP duration (894.5±36.92 versus 746.3±33.71, n=5, *P*=0.021). Wortmannin, a phosphatidylinositol 3‐kinase inhibitor, eliminated these VEGF‐induced effects. VEGF had no significant effect on the rapidly activating delayed rectifier potassium current (I_K__r_), resting membrane potential, AP amplitude, or maximal velocity of depolarization.

**Conclusions:**

VEGF inhibited I_K__s_ in a concentration‐dependent manner through a phosphatidylinositol 3‐kinase–mediated signaling pathway, leading to AP prolongation. The results indicate a promising therapeutic potential of VEGF in prevention of ventricular tachyarrhythmias under conditions of high sympathetic activity and ischemia.

**Clinical Perspective**

**What Is New?**

- Because the mechanisms of vascular endothelial growth factor (VEGF) on electrical properties of cardiomyocytes have not been fully elucidated, we investigated the direct effects of VEGF on delayed rectifier potassium current and action potential parameters.
- VEGF inhibited the slowly activating delayed rectifier potassium current in a concentration‐dependent manner through a phosphatidylinositol 3‐kinase–mediated signaling pathway, leading to action potential duration prolongation.
- VEGF had no significant effect on the rapidly activating delayed rectifier potassium current, resting membrane potential, action potential amplitude, or maximal velocity of depolarization.

**What Are the Clinical Implications?**

- The results show that stabilizing cardiac electrical activity may be 1 of the cardioprotective properties of VEGF and indicate a promising therapeutic potential of VEGF in prevention of ventricular tachyarrhythmias under conditions of high sympathetic activity and ischemia.

## Introduction

Biopharmaceutical‐based therapy of ischemic heart disease, especially gene therapy and stem‐cell therapy, shows promising results in animal and clinical studies.^1^ Vascular endothelial growth factor (VEGF), an angiogenic cytokine, plays an important role in these therapies^2^, ^3^, ^4^ through VEGF‐induced migration of stem cells to ischemic myocardium and neovascularization (including angiogenesis and arteriogenesis).^5^, ^6^, ^7^ However, studies show that VEGF might exert protective functions that extend far beyond its angiogenic activity and stem cell–mediated cardiac repair activity.^8^, ^9^ Protecting cardiomyocytes from apoptosis and imposing a positive inotropic effect on cardiomyocytes demonstrate that VEGF might have direct effects on cardiomyocytes, which may also contribute to its cardioprotection ability.^10^, ^11^

VEGF activates phosphatidylinositol 3‐kinase (PI3K) through binding to the VEGF type‐2 receptor and produces multitudinous functions.^12^, ^13^ PI3K‐mediated signaling has proved to be involved in the regulation of ion channels and cardiac action potential (AP) and plays an important role in antiarrhythmia.^14^, ^15^, ^16^ The delayed rectifier potassium current (I_K_) is the major outward current responsible for AP repolarization. Two components of the I_K_, a slowly activating delayed rectifier potassium current (I_Ks_) and a rapidly activating delayed rectifier potassium current (I_Kr_), have been identified in many mammalian species. Dysfunction of I_K_ is related to a change in action potential duration (APD) and may contribute to the creation of arrhythmia.^17^

Therefore, we hypothesized that VEGF exerts direct effects on I_K_ and other cardiac electrical properties, which are poorly understood. We investigated the effects of VEGF on I_K_ and AP parameters in guinea pig ventricular myocytes to explore the therapeutic potential and safety profiles of VEGF in arrhythmia and other cardiovascular diseases.

## Methods

The data, analytic methods, and study materials will be made available to other researchers for purposes of reproducing the results or replicating the procedure. The data that support the findings of this study are available from the corresponding author on reasonable request.

### Myocytes Isolation

Animal protocols used in this study were approved by the Institutional Animal Care and Use Committee of Zhengzhou University for Medical Research. Single ventricular myocytes were obtained from adult guinea pigs (female, 250‐350 g). Ventricular myocytes obtained from the first guinea pig were used for I_Ks_ and I_Kr_ recording; ventricular myocytes obtained from the second guinea pig were used for AP recording; ventricular myocytes obtained from the third guinea pig were used to investigate whether PI3K‐mediated signaling is related to VEGF‐induced effects. There were no repeated measurements on the same experimental unit. The isolation procedure is similar to the previously described method.^18^, ^19^ The heart excised from a guinea pig was initially placed on a Langendorff apparatus and perfused with Ca^2+^‐free Tyrode solution (in mmol/L: NaCl 136, KCl 5.4, MgSO_4_ 1.0, KH_2_PO_4_ 0.33, glucose 10, and HEPES 10; pH adjusted to 7.3‐7.4 with 1 mmol/L NaOH) for 5 minutes. Then the heart was perfused for 15 to 20 minutes with an enzyme solution (Ca^2+^‐free Tyrode solution including collagenase type II 0.5 g/L, pronase E 0.2 g/L, 2,3‐butanedione 2‐monoxime 0.5 g/L, carnitine 0.4 g/L, aurine 0.62 g/L, l‐glutamic acid 0.4 g/L). The left ventricle was dissected, minced, incubated, and stirred mechanically in potassium buffer solution (in mmol/L: KOH 80, KCl 20, l‐glutamic acid 50, MgCl_2_ 1, HEPES 10, glucose 10, KH_2_PO_4_ 20, taurine 20 and EGTA 0.5; pH adjusted to 7.3‐7.4 with 1 mol/L KOH). When the isolation was done well, rod‐shaped myocytes were visible under the microscope. The cells were filtered through a 200‐μm nylon mesh and resuspended in potassium buffer solution in which the calcium concentration was gradually increased to 1.0 mmol/L. The isolated myocytes were stored at room temperature (21°C to 25°C) until use.

### I_Ks_ and I_Kr_ Recording

Myocytes were placed in a recording chamber on the stage of an inverted microscope (Olympus IX71, Tokyo, Japan). The chamber was constantly superfused with extracellular solution (in mmol/L: NaCl 135, KCl 5.4, MgCl_2_ 1, NaH_2_PO_4_ 0.33, HEPES 10, glucose 10, BaCl_2_ 0.3, CdCl_2_ 0.2; pH was adjusted to 7.4 with NaOH) via a gravity‐fed solution delivery system. I_Ks_ and I_Kr_ were recorded in a whole‐cell single‐electrode voltage‐clamp configuration of the patch‐clamp technique using an EPC10 amplifier (Heka, Lambrecht/Pfalz, Germany). A computer equipped with PatchMaster (Heka) was used for generation of voltage‐clamp protocols and for data storage and evaluation. Patch electrodes had a resistance of 2 to 5 MΩ when filled with electrode internal solution (in mmol/L: KCl 120, MgCl_2_ 1, EGTA 5, phosphocreatine disodium salt 14, Na_2_‐ATP 5; pH was adjusted to 7.2 with KOH).

The I_Ks_ current traces were elicited in response to voltage steps from −40 to +60 mV in 10‐mV increments with a duration of 5 seconds, followed by a repolarized potential of −40 mV to test the tail current. The membrane potential was held at −40 mV. To record I_Ks_, E4031 (2 μmol/L) was added to the extracellular solution to block I_Kr_. The I_Kr_ current traces were elicited in response to the voltage steps from −60 to +60 mV in 10‐mV increments with a duration of 4 seconds, followed by a repolarized potential of −60 mV to test the tail current. The membrane potential was held at −60 mV. To record I_Kr_, chromanol 293B (30 μmol/L) was added to the extracellular solution to block I_Ks_.

### Single Myocyte AP Recording

APs were recorded in the whole‐cell, single‐electrode, current‐clamp configuration. The extracellular solution was composed of (in mmol/L) NaCl 140, KCl 3.5, HEPES 10, d‐glucose 10, NaH_2_PO_4_ 1.25, MgCl_2_ 1, CaCl_2_ 2; pH was adjusted to 7.4 with NaOH. The pipette was filled with a solution composed of the following (in mmol/L): K‐gluconate 140 mmol/L, NaCl 5, MgCl_2_ 1, CaCl_2_ 0.1, Mg‐ATP 2, HEPES 10, EGTA 1; pH was adjusted to 7.2 with KOH. APs were recorded in response to a 10‐ms current, which was determined by a leading current step from 0 to 900 pA in 100‐pA increments with a duration of 10 ms.

### Drug Application

Recombinant VEGF165 proteins (Sino Biological Inc, Beijing, China) of different concentrations (10, 40, 100, and 300 ng/mL) were included in the medium for 10 minutes during the dose‐response experiments. In some experiments isolated ventricular cardiomyocytes were pretreated with a PI3K inhibitor (100 nmol/L wortmannin, Selleck Chemicals, Houston, TX) for 1 hour before the addition of VEGF165 (100 ng/mL), and the inhibitor was present throughout the experiments in order to determine whether PI3K‐mediated signaling is involved in VEGF165‐induced inhibition of I_Ks_ and prolongation of APD.

### Statistical Analysis

The data were presented as mean±SEM and analyzed with SPSS 19.0 (SPSS Inc, Chicago, IL). Student t test or a 1‐way ANOVA followed by Bonferroni analysis for multiple comparisons were used to determine the statistical significance. A value of *P*<0.05 was considered statistically significant.

## Results

### Effects of VEGF on I_Ks_

**Figure 1:**
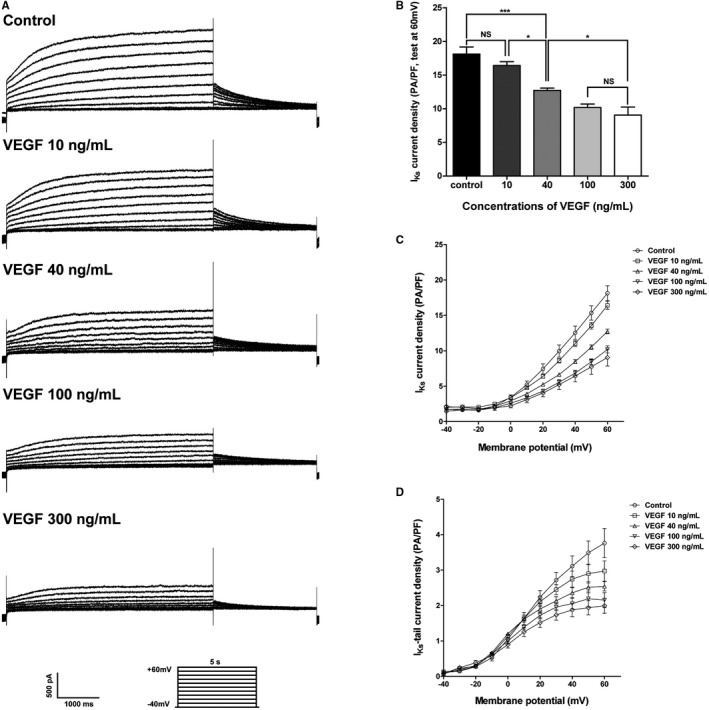
Effect of VEGF on I_K__s_ in isolated guinea pig ventricular myocytes (per dose sample size; n=5‐6). A, I_K__s_ recordings from the samples treated with or without 10, 40, 100, and 300 ng/mL VEGF. Current traces were elicited in response to the voltage steps from −40 to +60 mV in 10‐mV increments with a duration of 5 seconds. Holding potential was −40 mV. E4031 was added to isolate I_K__s_. B, Summarized data of I_K__s_ current density at +60 mV. *P<0.05, ***P<0.001, NS indicates not significant. C, The current‐voltage curves of I_K__s_ recorded in controls and in the presence of different concentrations of VEGF. D, The current‐voltage curves of I_K__s_‐tail recorded in controls and the presence of different concentrations of VEGF. I_Ks_ indicates slowly activating delayed rectifier potassium current; VEGF, vascular endothelial growth factor.

We initially tested the effects of different concentrations of VEGF on I_Ks_. Isolated guinea pig ventricular myocytes were treated with 10, 40, 100, and 300 ng/mL VEGF (Figure 1A). At +60 mV, the summarized data (n=5) of I_Ks_ current density (pA/pF) was 18.13±1.04 (control), 16.42±0.57 (10 ng/mL), 12.73±0.34 (40 ng/mL), 10.19±0.50 (100 ng/mL), 9.05±1.20 (300 ng/mL) (Figure 1B). At 10 ng/mL, VEGF treatment did not have significant effects on I_Ks_ compared with control group (*P*>0.05). There was also no significant difference between the 100 and 300 ng/mL VEGF treatment groups (*P*>0.05). Figures 1C and 1D show the current‐voltage curves of the I_Ks_ and I_Ks_‐tail under the control condition and using different concentrations of VEGF. I_Ks_ was significantly inhibited in a concentration‐dependent manner. The current‐voltage relationship was not affected.

### Effects of VEGF on I_Kr_

**Figure 2:**
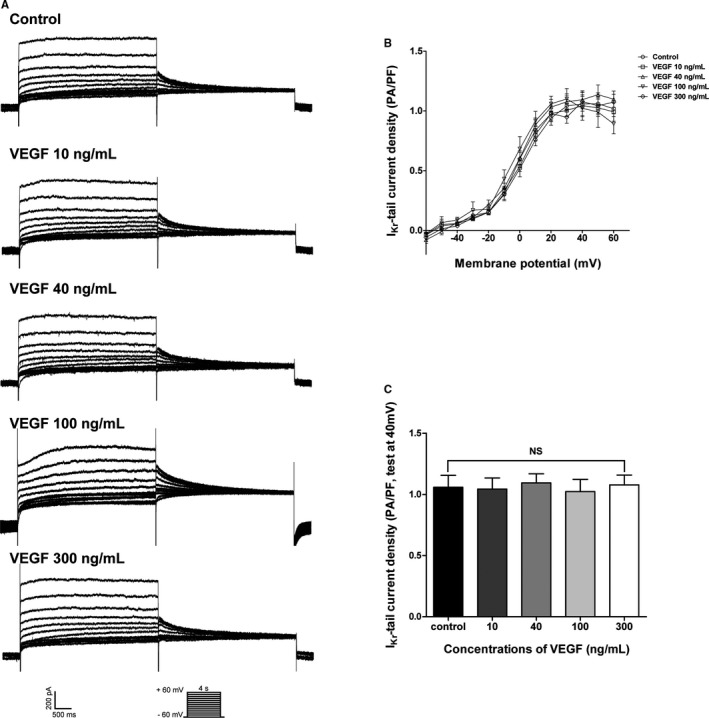
Effect of VEGF on I_K__r_ in isolated guinea pig ventricular myocytes (per dose; sample size n=5). A, I_K__r_ recordings from samples treated with or without 10, 40, 100, and 300 ng/mL VEGF. Current traces were elicited in response to voltage steps from −60 to +60 mV in 10‐mV increments with a duration of 4 seconds; holding potential was −60 mV. Chromanol 293B was added to isolate I_K__r_. B, The current‐voltage curves of the I_K__r_‐tail recorded in controls and in the presence of different concentrations of VEGF. C, Summarized data of I_K__r_‐tail current density at +40 mV. I_Kr_ indicates rapidly activating delayed rectifier potassium current; NS, not significant; VEGF, vascular endothelial growth factor.

We also tested the effects of VEGF on I_Kr_. Within the voltage range of −60 to +60 mV, a comparison of VEGF treatment groups with different concentrations showed no significant differences when compared with the control group, and no significant difference was observed between these VEGF treatment groups. In addition, the current‐voltage relationship was not affected (Figures 2A and 2B). At +40 mV, the summarized data (n=5) of I_Kr_‐tail current density (pA/pF) was 1.02±0.06 (control), 0.99±0.08 (10 ng/mL), 1.10±0.07 (40 ng/mL), 0.81±0.10 (100 ng/mL), and 1.07±0.07 (300 ng/mL) (Figure 2C).

### Effects of VEGF on the Activation of I_Ks_ and I_Kr_

**Figure 3:**
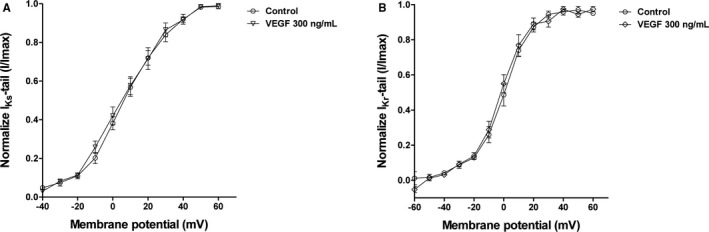
Effect of VEGF on the activation of I_K__s_ and I_K__r_ (per group; sample size n=5). A, Normalized current‐voltage relationships for the I_K__s_‐tail current (control group and 300 ng/mL treatment group). Curves are fits to experimental data by a Boltzmann function (V_1/2_: 7.646±1.310 mV vs 5.635±1.736 mV, *P*>0.05). B, Normalized current‐voltage relationships for the I_K__r_‐tail current (control group and 300 ng/mL treatment group). Curves are fits to experimental data by a Boltzmann function (V_1/2_: 0.006±0.920 mV vs −2.752±1.030 mV, *P*>0.05). I_Kr_ indicates rapidly activating delayed rectifier potassium current; I_Ks_, slowly activating delayed rectifier potassium current; VEGF, vascular endothelial growth factor.

Compared with the control group, the 300 ng/mL VEGF treatment group did not show an altered voltage‐dependent activation of the I_Ks_ and I_Kr_ channels. The half‐maximally activated potentials (V_1/2_) of I_Ks_ and I_Kr_ were not affected significantly (Figures 3A and 3B).

### Effects of Wortmannin on VEGF‐Induced I_Ks_ Inhibition

**Figure 4:**
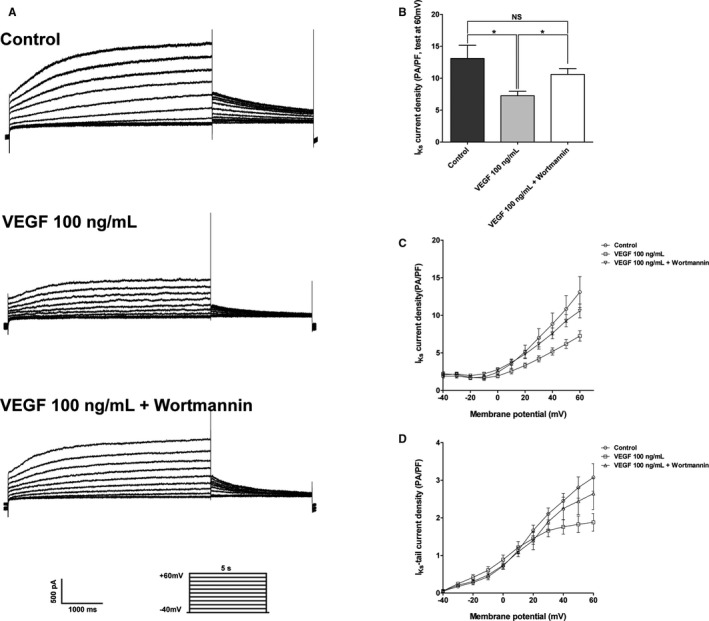
Effect of wortmannin on VEGF‐induced inhibition of I_K__s_ in isolated guinea pig ventricular myocytes (per group; sample size n=5). A, Recordings of I_K__s_ obtained from samples treated with the control condition, 100 ng/mL VEGF and 100 ng/mL VEGF plus wortmannin. B, Summarized data of I_K__s_ current density at +60 mV. *P<0.05, NS indicates not significant. C, The current‐voltage curves of I_K__s_ for the control group, 100 ng/mL VEGF group and 100 ng/mL VEGF plus wortmannin group. D, The current‐voltage curves of the I_K__s_‐tail for the control group, 100 ng/mL VEGF group and 100 ng/mL VEGF plus wortmannin group. I_Ks_ indicates slowly activating delayed rectifier potassium current; NS, not significant; VEGF, vascular endothelial growth factor.

We used wortmannin, a PI3K inhibitor, to study whether PI3K mediated signaling is related to VEGF‐induced I_Ks_ inhibition. Figure 4A shows the representative recordings of I_Ks_ in isolated guinea pig ventricular myocytes treated with the control condition, 100 ng/mL VEGF and 100 ng/mL VEGF plus wortmannin (100 nmol/L). Figures 4C and 4D show the current‐voltage curves of I_Ks_ and I_Ks_‐tail for the control group, 100 ng/mL VEGF group, and 100 ng/mL VEGF plus wortmannin group. There was no significant difference in the amplitudes of I_Ks_ between the control and those recorded in the presence of 100 ng/mL VEGF plus wortmannin. Wortmannin eliminated the VEGF‐induced I_Ks_ inhibition.

### Effects of VEGF on AP Parameters

**Figure 5:**
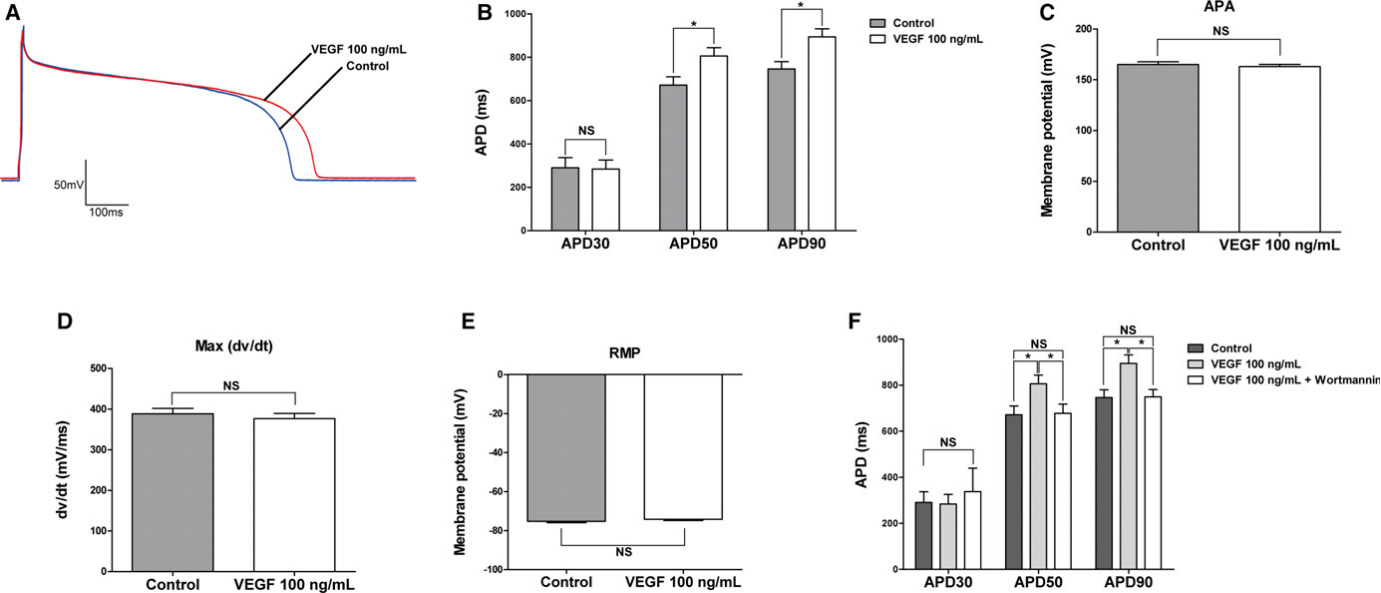
Effect of 100 ng/mL VEGF on action potential parameters in single cardiac ventricular myocytes (per group; sample size: n=5). A, Recordings of action potential in the control and in the presence of 100 ng/mL VEGF in isolated guinea pig ventricular myocytes. B through E, Summary of the effects of 100 ng/mL on APD (B), APA (C), Max (dv/dt) (D), and RMP (E). F, Summarized data of APD for the control group, 100 ng/mL VEGF group and 100 ng/mL VEGF plus wortmannin group. Values are mean±SEM. APA indicates action potential amplitude; APD, action potential duration; Max (dv/dt), maximal velocity of depolarization; RMP, resting membrane potential; VEGF, vascular endothelial growth factor. *P<0.05, NS, not significant.

To investigate the effects of VEGF on the cell membrane potential, isolated guinea pig ventricular myocytes were treated with 100 ng/mL VEGF. Figure 5A shows the AP waveforms of the 100 ng/mL VEGF treatment group and the control group. APD at 50% repolarization (APD50) and 90% repolarization (APD90) were prolonged (APD50: 805.8±38.04 ms versus 671.3±38.61 ms, n=5, *P*=0.045; APD90: 894.5±36.92 ms versus 746.3±33.71 ms, n=5, *P*=0.021), and there were no significant differences in APD30 (283.9±41.84 ms versus 290.6±46.64 ms, n=5, *P*>0.05), resting membrane potential (−75.25±0.65 mV versus −74.17±0.59 mV, n=5, *P*>0.05), AP amplitude (164.9±2.71 mV versus 162.9±2.17 mV, n=5, *P*>0.05), or maximal velocity of depolarization (388.6±13.17 mV/ms versus 376.8±12.72 mV/ms, n=5, *P*>0.05) between the 100 ng/mL VEGF treatment group and the control group (Figures 5B through 5E). Figure 5F shows the APD for the control group, the 100 ng/mL VEGF group, and the 100 ng/mL VEGF plus wortmannin group. Wortmannin eliminated the VEGF‐induced prolongation of APD50 (100 ng/mL VEGF group versus 100 ng/mL VEGF plus wortmannin group: 805.8±38.04 ms versus 678.1±39.75 ms, respectively; n=5, *P*=0.028) and APD90 (100 ng/mL VEGF group versus 100 ng/mL VEGF plus wortmannin group: 894.5±36.92 ms versus 749.1±32.02 ms, respectively; n=5, *P*=0.020).

## Discussion

The key observations of the present study are as follows. First, VEGF inhibited I_Ks_ in a concentration‐dependent manner in guinea pig ventricular myocytes. I_Ks_ was not further inhibited by 300 ng/mL VEGF, probably indicating that the VEGF cell membrane receptors were saturated by 100 ng/mL VEGF (Figure 1C). Second, VEGF at different concentrations did not have a significant effect on I_Kr_. Third, VEGF did not alter the activation of I_Ks_ and I_Kr_. Fourth, VEGF prolonged APD50 and APD90 at the concentration of 100 ng/mL but had no effect on other AP parameters. Fifth, a PI3K inhibitor eliminated VEGF‐induced inhibition of I_Ks_ and APD prolongation, suggesting that VEGF induced its effects mainly in a PI3K signaling–dependent mechanism.

AP repolarization is an important phenomenon in cardiac myocytes. I_K_, composed of I_Kr_ and I_Ks_, acts as a major outward current during repolarization, blockage of which leads to delayed repolarization and the prolongation of APD. I_Ks_ is the major outward current during the period of plateau repolarization.^20^ Therefore, VEGF‐induced APD prolongation may partially be attributed to inhibition of I_Ks_. In addition, the L‐type calcium current, an inward current during the plateau phase of repolarization, also affects the APD. Our previous studies have found that VEGF increased L‐type calcium current in guinea pig ventricular myocytes.^21^ Therefore, VEGF‐induced enhancement of L‐type calcium current may also be 1 of the mechanisms underlying VEGF‐induced APD prolongation.

Most class III antiarrhythmic drugs are highly selective I_Kr_ channel blockers and lengthen APD in a reverse frequency‐dependent manner.^22^, ^23^ These drugs have little APD‐prolonging effect under the condition of a high heart rate, limiting their therapeutic potency against tachyarrhythmias. In addition, APD prolongation caused by blocking I_Kr_ exhibits a proarrhythmic propensity, increasing the risk of QT prolongation and Torsades de Pointes.^24^ Blocking of I_Ks_ does not show a reverse frequency dependence, suggesting that the blocking of I_Ks_ shows more potential for preventing tachyarrhythmias.^23^, ^25^, ^26^ I_Ks_ has been shown to increase under high β‐adrenergic stimulation because of the slow deactivation kinetics during negative potentials.^22^, ^27^ Studies show that high sympathetic nerve activity easily facilitates malignant ventricular tachyarrhythmias.^28^, ^29^ Blocking I_Ks_ can prevent proarrhythmic effects under the condition of high sympathetic tone, suggesting that enhancement of I_Ks_ may play a pivotal role in ventricular proarrhythmia under high sympathetic activity.^30^

With regard to the treatment of ischemic heart disease, especially myocardial infarction, VEGF has shown great potential for cardioprotection, including attenuating infarct size, preventing ventricular remodeling and improving cardiac function.^3^, ^4^, ^31^ In addition, it should be noted that VEGF gene‐based therapy for ischemic heart disease has been shown to avoid serious or lethal arrhythmias.^8^ This finding suggests that stabilizing cardiac electrical activity is also an important part of VEGF‐induced cardioprotection. It is known that sympathetic overactivity is closely associated with the incidence of malignant arrhythmias,^32^, ^33^ in which increasing I_Ks_ may be involved. Therefore, inhibition of I_Ks_ probably is the underlying mechanism in prevention of malignant ventricular arrhythmias. However, because I_Ks_ blockage contributes to long‐QT syndrome,^34^ more extensive studies are needed to investigate the safety profiles of VEGF in the treatment of arrhythmia and ischemic heart disease. This future research will help predict the direct clinical outcome of I_Ks_ inhibition by VEGF.

It is known that the I_Ks_ channel is composed of α subunits and function‐altering β subunits, and the I_Kr_ channel is composed of HERG and unestablished β subunits.^17^ Studies show that the β subunit of I_Ks_ plays an essential role in acute I_Ks_ inhibition, in which PI3K‐mediated signaling is involved.^35^ This finding may explain why VEGF inhibited I_Ks_ but had no effect on I_Kr_.

## Sources of Funding

This work was supported by National Natural Science Foundation of China (grant number U1604184).

## Disclosures

None.
